# The Question of General or Local Anesthesia1Read at the Annual Thirty-sixth Meeting of the Medical Association of Central New York, at Buffalo, October, 1905.

**Published:** 1906-02

**Authors:** E. S. Vanduyn

**Affiliations:** Syracuse, N. Y.; Lecturer on Surgery, Syracuse University; Assistant-Surgeon Hospital of Good Shepherd


### The Question of General or Local Anesthesia.[1]

By E. S. VANDUYN, B. S., M. D., Syracuse, N. Y.,

Lecturer on Surgery, Syracuse University; Assistant-Surgeon Hospital of Good Shepherd.

THE dangers of general anesthesia are known to everyone accustomed to their use. That these dangers are present in every administration of ether or chloroform is recognised by every surgeon and thought is given by them as to the condition in

1. Read at the Annual Thirty-sixth Meeting of the Medical Association of Central New York, at Buffalo, October, 1905.

general of the patient, but especially to that of the circulatory and respiratory systems. Yet, where an operative procedure has been decided upon, seldom does the presence of some untoward condition cause more than greater care to be employed in the administration of the anesthetic; and perhaps more careful attention and time is given to the preparation of the patient for anesthetization. I say "seldom", because we all know how few of those entering hospitals for whom operative procedures are indicated, escape the operation. If we should review the yearly record of hospital operations, we would find moreover that general anesthesia was the only and routine means employed, and in review of a year's work we should surely find extremely few operations, outside of minor dressings and simple incisions, done with local anesthesia. Whether this is because the race is becoming more sensitive to pain and proportionately anxious to escape it, or whether routine on the part of the surgeon, and desire to make the operative procedure the least possible offensive to the patient, and therefore attractive as possible, is responsible for the universal employment of a general anesthetic, I am not sure but feel that both are responsible factors. While the surgeon is conscious of the dangers attending the administration of a general anesthetic, so seldom does immediate death follow its use, that he is content to make its administration as safe as it is possible for him to do, by careful preparation of the patient, the employment of a skilled anesthetist, and rapid operating, but is unwilling to avail himself of the opportunity to escape entirely from the possibilities of even these dangers, afforded by the use of a local anesthetic. Instead of relegating local anesthesia to the domain of minor surgery, the existence of such a means to perform operations without great pain to the patient, and still escape the dangers attending the employment of inhilation anesthesia should be received as a great advance in conservative surgery. It should be employed whenever possible and should be regarded as imperative where the condition of the patient is any way below the best possible condition as regards the safe administration of a general anesthetic. Besides the danger of immediate death through effect on the heart or respiration, or the extension of their depressing effects to the vital centers in the medulla, there are many other untoward after effects directly traceable by careful analysis to the poisonous effects of ether or chloroform. The excretion of these drugs into the stomach increases the toxicity of the stomach contents, produces reflex atony of the stomach and intestines and accumulation of gases, and the restraint over any toxic producing substances present in the alimentary tract is lessoned. The functionating power of the various excretatory organs is lowered; the elimination of toxic substances is diminished; the normal chemical balance of the blood serum is upset and there is a loss of some of its resistance to the action of the normal bacterial toxins and pathogenic micro-organisms. Cell metabolism is interferred with and modified and may produce toxic products causing auto-intoxication with the formation of hemolytic and other injurious substances. While no apparantly serious harm usually results, the presence of these conditions is directly demonstrable. And, on the other hand, they often do serious after troubles which are commonly laid to other causes. Chloroform and ether unquestionably increase post-operative shock and prolong depression. They retard repair and decrease the power of resistance to infection. We may confidently look forward to a time, not far distant, when physiological chemistry will give the exact formula the net damage to cell and function, the result of such profound interference. Especially is serious trouble liable to result from these conditions accompanying the administration of a general anesthetic in the old and very young, through their deficiency of recuperative power. Children who give a history of repeated billious attacks, often develop severe auto-intoxication from alimentary disturbances which follow the administration of ether or chloroform and in direct proportion to the amount of the anesthetic inhaled.

Many times it is more reasonable to explain post-operative inflamations as caused indirectly by the general anesthesia than to assume a secondary infection to have occurred in spite of the most rigid aseptic precautions on the part of the operator.

From time to time I have been struck with the success obtained through the employment of local anasthesia in these classes of cases as regards the freedom from these after effects of ether and chloroform, and in illustration I will cite briefly a few of these cases with which I have had personal connection.

The first case is that of a middle-aged woman advanced in pulmonary tuberculosis. Her attending physician had diagnosed tubercular peritonitis. The abdomen was greatly distended and tender. A careful review of the history and present condition made the diagnosis as made, seem probable. Relief from pressure was urgently indicated. On account of the feeble condition of the patient and the necessity of a simple incision only, the incision was made after local use of cocaine. The line of incision was three and one-half inches long. The patient said she felt no pain in the making of this incision which was through the parietal peritoneum. Then it was seen that we had to do with a large ovarian cyst which was exposed. The cyst was punctured and its contents evacuated without discomfort. After the sac had been emptied it was readily drawn out through the wound. To this point there was not the least pain but tension on the pedicle and its ligation caused a sickening sensation that made the patient cry out. This was the only part of the operation in which the patient complained of pain. General anesthesia being feared, operation was planned under cocaine for the relief of what was supposed to be ascites and to gain the unexplained benefit that so often comes from incision in tubercular peritonitis. With the completion of the ovariotomy, recovery occurred without any depression or increased activity in the tubercular process in the lungs. This occurred early in my experience and opened my eyes both to the importance of the use of local anesthesia and the possibility of its employment in major operations of almost any degree.

The second case was that of an old man in his seventy-ninth year, who was brought into the hospital with an old inguinal hernia that had been strangulated for a day and a half. The man was extremely weak and exhausted and almost continuously delirious. Operation was performed under cocaine anesthesia and was completed in seven minutes. The sac was opened and its contents having been returned to the abdomen, was ligated and stitched up behind the ring; the ring was then sutured with deep mattress stitches and the superficial wound closed with continuous suture. All was without pain or complaint from the patient. There seemed no additional shock. The man recovered rapidly and in two or three days, to all appearances, was as strong and well as ever.

The third case was that of a large thyroid cyst in a man thirty-nine years old that caused marked paroxisms of dyspnoea. He was extremely nervous and had become greatly enfeebled. The left lobe of the thyroid with its cyst was removed under cocaine anaesthesia, fifteen minutes being required for the operation. During the operation, injections into the deeper tissues were employed twice. The operation was performed with the man seated in a chair to avoid the reclining position that caused difficulty in breathing. He joked and told stores through the entire operation, to the wonderment of all. Immediate relief from the symptoms, rapid recovery from the operation, and freedom from infection were the satisfactory results.

The fourth case was that of a man eighty-four years old, with strangulated right inguinal hernia. He was in an unusually feeble condition. Operation under a general anesthesia had been advised against on account of the almost certainty that the patient would not survive the ordeal. Incision was made under cocaine anesthesia and the hernia reduced. Relief from the distressing symptoms was immediate with the performance of the operation, and in two or three days the patient had recovered his usual strength.

I want to call attention to one other important point in these cases, that although the patients were many of them old and feeble, and their circulation low, in none did suppuration in the wound occur and repair was always good and prompt. Moreover, in the greater number of these cases, what was the most striking, the exigencies of the operation being such as to preclude long and efficient preparation of the field of operation, there followed no subsequent suppuration, and I became impressed with the idea that the freedom from infection was due to the fact that (the patient's vitality not reduced by the effects of the general anaesthetic) the cell activity and resistance in the field of injury were not diminished, and the general resistance to bacterial inroads was reinforced by the immediate relief from pain and the other dstressing symptoms.

In closing I will cite two cases that especially illustrate this point. Both cases were feeble, old persons over eighty years of age.

The Rev. A, eighty-three years old, with a large cutaneous carcinoma on the right forearm, beginning to break down and presenting a large ulcerating surface, was removed under local anaesthesia. The skin was brought together with subcutaneous suture and healed by first intention. The last case is that of an old lady eighty-four years old, who had carried a large lipoma, seven inches in diameter, on her back for twenty-five years. She had recently begun to complain of severe pain and increase in size of the tumor. It was evident that an inflammatory action was going on in the tumor, due to the irritation of the chair-back, the poorer circulation of advancing years, and also probably to a few recent x-ray treatments that she had submitted to. A vertical incision five inches in length was made over this tumor under cocaine anaesthesia. The mass was found to be undergoing inflammatory change and was broken down to a considerable extent, yet the wound united quickly without pus formation and was entirely healed on the tenth day.

Before anesthesia, alcohol and opium played a great role in the mitigation of suffering during operation and naturally was ineffective. Upon the discovery of ether and chloroform, the pendulum swung naturally to the extreme limit the other way, from partially obtunded senses to total unconsciousness, and there it still remains. In the present paper I do not pretend to give a scientific proof of a theory, nor do I venture to insist on a practice from the results observed in so few and inconclusive cases, but it is offered only as a brief clinical statement with its more obvious deductions.

318 JAMES STREET.

